# Diversified humoral immunity and impacts of booster vaccines: SARS-CoV-2 antibody profile and Omicron BA.2 neutralization before and after first or second boosters

**DOI:** 10.1128/spectrum.00605-24

**Published:** 2024-08-20

**Authors:** Xiaochun Susan Zhang, Anne Windau, Jamie Meyers, Xiaohua Yang, Feng Dong

**Affiliations:** 1 Department of Pathology, University Hospitals Cleveland Medical Center, Case Western Reserve University, School of Medicine, Cleveland, Ohio, USA; 2 College of Medicine, Northeast Ohio Medical University, Rootstown, Ohio, USA; MultiCare Health System, Tacoma, Washington, USA

**Keywords:** SARS-CoV-2, vaccine, booster, antibody profile, neutralizing antibodies, hybrid immunity

## Abstract

This study aims to investigate humoral immunity against severe acute respiratory syndrome coronavirus 2 (SARS-CoV-2) and assess the impact of booster vaccines. We recruited individuals scheduled to receive either the first (original formula) or the second (bivalent) booster following the initial two-dose SARS-CoV-2 vaccination. We tested for IgG antibodies targeting the spike protein receptor-binding domain (RBD), S1, S2, and nucleocapsid protein, as well as for neutralizing antibodies against Omicron BA.2, before and 14–28 days after receiving the boosters. One year after receiving the initial series of vaccinations, all participants maintained anti-RBD/S1 antibodies. However, levels were lower in individuals who were vaccinated only compared to those who had both vaccination and prior infection (hybrid immunity). Participants with hybrid immunity also showed higher retention of neutralizing antibodies (93% compared to 24% in vaccine-only individuals). Even before receiving any booster shots, participants with hybrid immunity had antibody levels similar to those of vaccine-only individuals after their first booster. After receiving booster shots, antibody levels at 14–28 days were similar regardless of the number of boosters or the type of immunity. About 1 year after the first booster, all participants maintained neutralizing antibodies, and vaccine-only individuals retained about 10 times higher levels of binding antibodies than those without a booster. Humoral immunity varies widely among individuals, and vaccination planning should consider both vaccination and infection history. Boosters are beneficial for increasing antibody levels to ensure sufficient protection against infection and helping bridge the immunity gap between vaccine-only and hybrid immunity.

**IMPORTANCE:**

As we move into the era of severe acute respiratory syndrome coronavirus 2 (SARS-CoV-2) vaccine boosters and shifting from pandemic to endemic, the landscape has changed for both the circulating SARS-CoV-2 variants and population immunity. Even though recent waves of infection have been clinically milder than earlier variants due to the high levels of population immunity and the properties of the Omicron subvariants, vaccination remains crucial for managing COVID-19 in the post-pandemic era. Our study unveils significant variations in the retention of anti-SARS-CoV-2 binding antibody profiles and neutralizing antibody levels 1 year after the primary and the first booster mRNA vaccination. It adds new information regarding how boosters change antibody levels and durability in individuals with hybrid (vaccination plus infection) or vaccine-only (never-infected) immunity. The findings can shed light on future vaccination planning.

## INTRODUCTION

Since 11 March 2020, WHO officially declared the severe acute respiratory syndrome coronavirus 2 (SARS-CoV-2) outbreak a pandemic; more than 776 million SARS-CoV-2 infections have been reported to date worldwide, according to the WHO COVID-19 dashboard (https://data.who.int/dashboards/covid19/cases?n=c). SARS-CoV-2 infection has resulted in more than 7 million deaths. Even though mass vaccination helped slow down the spread and protect individuals from severe disease and death, shifted from pandemic to endemic, SARS-CoV-2 is likely to stay, frequently mutate, and pose a constant threat globally.

Vaccination remains the most powerful tool in preventing SARS-CoV-2 infection, hospitalization, and death caused by COVID-19. New vaccines with updated immunogens have been released in response to the evolving prevalence of SARS-CoV-2 strains. In the U.S., on 19 November 2021, the Advisory Committee on Immunization Practices (ACIP) recommended that all persons aged ≥18 years receive a booster dose after the minimum recommended interval since the completion of primary vaccination (1). In October 2022, ACIP and the Centers for Disease Control and Prevention recommended that all persons aged ≥5 years receive one bivalent (Omicron BA.4/BA.5 strains) mRNA booster dose ≥2 months after the completion of any FDA-approved or FDA-authorized monovalent primary series or monovalent booster doses (2). However, by 31 December 2023, only 28% of the global population and 36% of the United States population had received one or more booster doses (https://data.who.int/dashboards/covid19/vaccines?n=c).

As we move into the era of SARS-CoV-2 vaccine boosters and shifting from pandemic to endemic, the landscape has changed for both the circulating SARS-CoV-2 variants and population immunity. Due to vaccinations and widespread and natural infections, population immunity has evolved from a simple vaccine- or infection-induced immunity to a more complex status with considerable variability. This immunity continues to diversify with subsequent infections and additional booster doses. Diversified humoral immunity plays a critical role in combating evolving SARS-CoV-2 variants by broadening the scope of antibody recognition, enhancing neutralization capacity, and providing more comprehensive and durable protection against infection and disease. However, the varied immunity among populations presents challenges for future vaccination strategies, especially when dealing with new SARS-CoV-2 variants. Studies have shown that individuals with hybrid immunity (a combination of vaccination and natural infection) have stronger and longer-lasting protection (3). This raises the question of whether the interval for booster vaccinations can be extended for those with hybrid immunity compared to those who have never been infected. Additionally, it is not clear to what extent individuals who have only been vaccinated (no prior infection) benefit from a yearly booster vaccine. Understanding the current status of SARS-CoV-2 antibody profiles, neutralizing antibody levels in the population, and how boosters change antibody levels and durability is essential for future vaccination strategies. This study aimed to investigate binding antibodies against various SARS-CoV-2 antigens and neutralizing antibody levels against the Omicron BA.2 strain, from which many recent variants originated, in immunocompetent healthcare workers before and after receiving the third (first booster) and fourth (second booster) doses of mRNA vaccines. Samples were collected before and after booster administration to determine residual antibody levels before the booster and assess post-booster changes in antibody profile. The study evaluated binding antibodies against spike protein (S) RBD, S1, and S2, as well as neutralizing antibodies to provide a comprehensive understanding of humoral immunity. Furthermore, anti-nucleocapsid (N) protein antibody and history of SARS-CoV-2 PCR test results were incorporated into differentiating vaccine-only versus hybrid immunity, providing further insight into the immune response.

## MATERIALS AND METHODS

### Study design and subjects

This study includes two cohorts, and participation was voluntary. Cohort 1 comprised 24 healthcare workers who were scheduled to receive the third dose of the original mRNA vaccine. Cohort 2 comprised 21 healthcare workers who had received three doses of the original vaccine and were scheduled to receive the fourth dose (bivalent vaccine). The inclusion criteria are as follows: (i) 18 years and older and a healthcare worker at UHHS, (ii) have received two doses of COVID vaccine at least 6 months ago, and (iii) eligible and plan to receive the third or fourth dose. The exclusion criteria are having an acute illness or being immunocompromised because of diseases or immunosuppressants. The study requested participants to provide informed consent, complete a survey, and have two study visits. During the first visit, which was within 1 week prior to the administration of their third or fourth dose of COVID-19 vaccine, we collected pre-3 or pre-4 vaccine samples for cohort 1 and cohort 2 participants, respectively. During the second visit, which was 14–28 days after receiving the vaccine, we collected the post-3 or post-4 vaccine samples. We used REDCap (Research Electronic Data Capture), a secure web application, to administer informed consent forms electronically, manage the surveys, and organize COVID-19 antibody test results.

### Specimen collection and processing

During each visit, 2–5 mL of blood from the participants was collected into a serum separator tube via venipuncture by a qualified phlebotomist. Samples were allowed to clot at room temperature for 30 minutes and then centrifuged at 3,000 rpm for 6 minutes. After centrifugation, serum samples were transferred into new tubes and stored at −80°C until testing.

### Measure SARS-CoV-2 binding antibodies using a multiplex immunoassay

Bioplex 2200 semi-quantitative SARS-CoV-2 IgG multiplex panel (Catalog number 12014192; Bio-Rad, California) was performed as per the manufacturer’s instructions. The assay detects and differentiates antibodies against the S RBD, S1, S2, and nucleocapsid protein IgG antibodies for RBD, S1, and nucleocapsid are traceable to the WHO international standard for anti-SARS-CoV-2 immunoglobulin (NIBSC 20/136). NIBSC 20/136 was established using a pool of convalescent plasma from 11 individuals who recovered from SARS-CoV-2 infection. For binding antibody assays, the assigned value for NIBSC 20/136 was 1,000 binding antibody units/mL (4). The assay cutoff for positivity is 12, 21, 9, and 23 U/mL for RBD, S1, S2, and nucleocapsid IgG antibodies, respectively.

### Measure neutralizing antibodies using surrogate virus neutralization test

Neutralizing antibodies against BA.2 were measured using the SARS-CoV-2 Surrogate Virus Neutralization Test Kit (GenScript, New Jersey) with modifications. The assay semi-quantitatively measures antibodies that block the interaction between the viral spike protein RBD and the ACE2 cell surface receptor. First, the samples and controls were pre-incubated with the horseradish peroxidase (HRP)-Omicron BA.2 RBD at 37°C for 30 minutes to allow the binding of neutralization antibodies to the HRP-RBD. The mixture was then added to the capture plate, which was pre-coated with the human ACE2 protein. Tetramethylbenzidine (TMB) solution was used for color development. The absorbance at 450 nm of the sample was read using a microplate reader. After subtracting background optical density (OD), 20% or more signal inhibition was considered neutralizing antibody-detectable. SARS-CoV-2 (Omicron) Neutralizing Antibody Standard (GenScript, New Jersey) was used to generate calibrators for quantitation.

### Statistical methods

The statistical analyses were conducted using the SigmaPlot software (Version 15.0, Grafiti LLC, Palo Alto, CA). For numerical data, the Shapiro–Wilk normality test was first performed to determine data distribution. If the normality test passed (i.e., normal distribution), the Student *t*-test and the one-way ANOVA were used to assess the significance of the difference between two groups and among three or more groups, respectively. If the normality test failed (i.e., non-Gaussian distribution), the Mann–Whitney rank sum test (a nonparametric test) was used to assess the significance between two groups, and the Kruskal–Wallis one-way ANOVA on ranks (nonparametric) was used to evaluate significance among three or more groups. The software automatically runs the normality test first and then performs the appropriate comparison test. Spearman rank order correlation was employed to evaluate the correlation between variables such as anti-RBD and neutralizing antibodies. A *P*-value of <0.05 was considered statistically significant. EP Evaluator (version 11.1, Data Innovations, Colchester, VT) was used to calculate central 95% intervals.

## RESULTS

A total of 69 participants were enrolled. Among them, 24 participants could not obtain paired pre- and post-vaccination samples and were excluded. The demographic characteristics of the study sample are summarized in Table 1. All the participants received their full series of primary mRNA vaccines (two doses) before enrollment. Prior to receiving their booster dose, 58% of cohort 1 and 48% of cohort 2 had a history of natural infection, resulting in an overall hybrid immunity rate of 53% among participants. Natural infection was determined by either a self-reported positive PCR test or a positive anti-nucleocapsid antibody result (it does not include self-administered antigen tests). The positive rate of anti-nucleocapsid antibody was 40% (18 out of 45). The participants’ self-reported PCR-positive rate was 31% (14 out of 45), and 9% (4 out of 45) of participants had positive PCR results more than one time. Notably, 89% positive PCR (16 out of 18) happened after receiving the two doses of primary vaccines based on the self-reported vaccination and PCR-positive dates. Sixty-seven percent of the positive PCR results happened between 1 November 2021 and 31 January 2022. As of 5 December 2020 (before vaccination), the seropositive rate of healthcare workers in UHHS was only 3.9% (5). Therefore, most natural infections in the study participants were breakthrough infections that occurred during the winter of 2021. Additionally, six participants (43%) reporting positive PCR results were negative for anti-nucleocapsid antibodies.

**TABLE 1:** Study cohorts and sample characteristics

	Cohort 1: pre- and post-3rd doses of the original vaccine (*n* = 24)	Cohort 2: pre- and post-4th doses of the bivalent vaccine (*n* = 21)
Age	Median: 41; range: 24–58 years old	Median: 48; 26–66 years old
Sex	Female: 23; male: 1	Female 16; male 5
Race	Non-white: 8; white: 16	Non-white: 4; white: 16; 1 not disclosed
Numbers of chronic diseases (diabetes, obesity, chronic kidney and liver disease, and stroke)	4 (16.7%); 2 had hybrid immunity	4 (19%); 1 had hybrid immunity
Booster mRNA vaccine	21 received Moderna; 2 received Pfizer; 1 unspecified	3 received Moderna; 17 received Pfizer; 1 unspecified
History of positive PCR	12 (4 had one positive PCR; four had two positive PCR tests at different times)	6 (all with one positive PCR test)
Anti-nucleocapsid IgG-positive rate	12 (50%)	6 (29%)
Natural infection rate (PCR or positive anti-nucleocapsid)	14 (58%)	10 (48%)
Pre-booster sample: time after the last dose of vaccine	Median: 376; range: 161–439 days; IQR (275–395 days)	Median: 353; range: 45–438 days; IQR (290–370 days)
Post-booster sample: time after the booster dose	Median: 16; range: 14–25 days	Median: 17; range: 14–24 days

Most subjects had an interval of about 300–400 days between their previous and booster dose. The mean intervals were 325, 349, 332, and 308 days for the pre-3 vaccine-only, pre-3 hybrid immunity, pre-4 vaccine-only, and pre-4 hybrid immunity participants, respectively (*P* > 0.05). Additionally, eight participants (four in cohort 1 and four in cohort 2) reported having chronic diseases such as diabetes, hypertension, heart disease, chronic kidney disease, and stroke. Among them, two of cohort 1 and one of cohort 2 participants had hybrid immunity.

**Fig 1:**
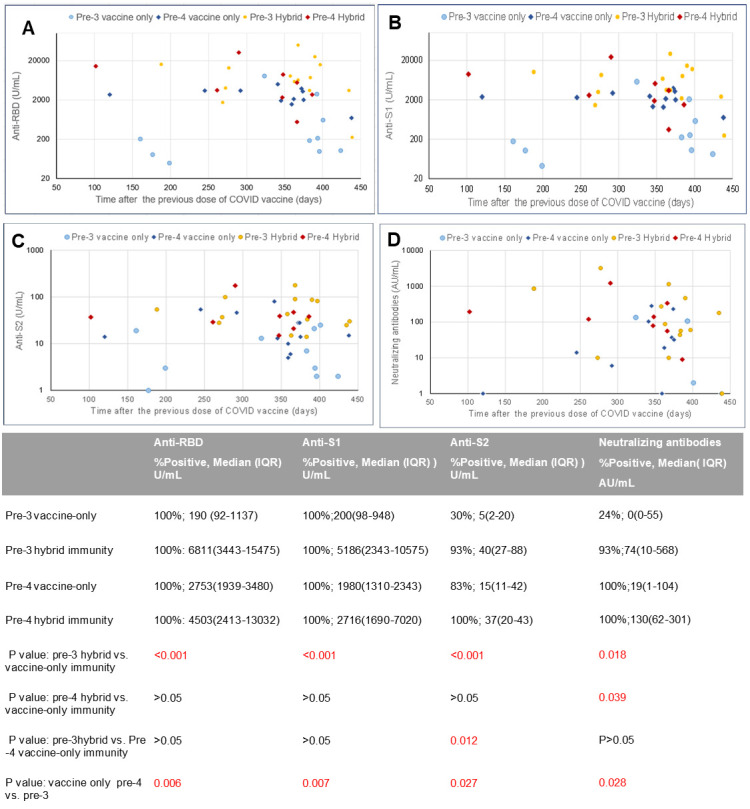
Remaining SARS-CoV-2 binding and neutralizing antibodies before receiving booster vaccines. (**A**) Anti-RBD binding antibodies before the third (first booster) or fourth (second booster) dose of mRNA vaccine by days after the previous vaccine. (**B**) Anti-S1 binding antibodies before the third (first booster) or fourth (second booster) dose of mRNA vaccine by days after the previous vaccine. (**C**) Anti-S2 binding antibodies before the third (first booster) or fourth (second booster) dose of mRNA vaccine by days after the previous vaccine. (**D**) Neutralizing antibodies before the third or fourth mRNA vaccine dose by days after the previous vaccine. The table at the bottom summarizes neutralizing and binding antibody levels and *P*-values between groups. Pre-3: prior to the third dose of the original mRNA vaccine. Pre-4: prior to the fourth dose of the bivalent mRNA vaccine. “Hybrid” denotes hybrid immunity due to the combination of vaccination and natural infection. “Vaccine-only” means the participants had no evidence of natural infection. IQR, interquartile range.

The remaining antibody levels before receiving booster vaccines (dose 3 or 4) are shown in Fig. 1. All cohort 1 (pre-third dose) participants were positive for anti-RBD and anti-S1 binding antibodies with concentrations ranging from 49 to 49,100 U/mL before receiving the third dose of vaccine regardless of their infection status. Additionally, 63% (20% of vaccine-only and 93% of hybrid immunity) participants showed anti-RBD and anti-S1 antibodies greater than 1,000 U/mL, which exceeded the WHO standard NIBSC 20/136 prepared from convalescent plasma. However, only 30% and 24% of vaccine-only individuals were positive for anti-S2 and neutralizing antibodies, respectively. Those with hybrid immunity showed significantly higher binding and neutralizing antibody levels than their vaccine-only counterparts. Cohort 2 (pre-fourth dose) vaccine-only participants showed substantially higher residual binding and neutralizing antibodies than cohort 1 vaccine-only participants. Their anti-RBD, anti-S1, and neutralizing antibodies were similar to those of cohort 1 participants with hybrid immunity. Cohort 2 participants with hybrid immunity showed no difference in binding antibodies but elevated neutralizing antibody levels compared to their vaccine-only counterparts. Before receiving the fourth dose, 86% of cohort 2 (83% vaccine-only and 89% hybrid immunity) participants had anti-RBD and anti-S1 antibody levels exceeding 1,000 U/mL.

**Fig 2:**
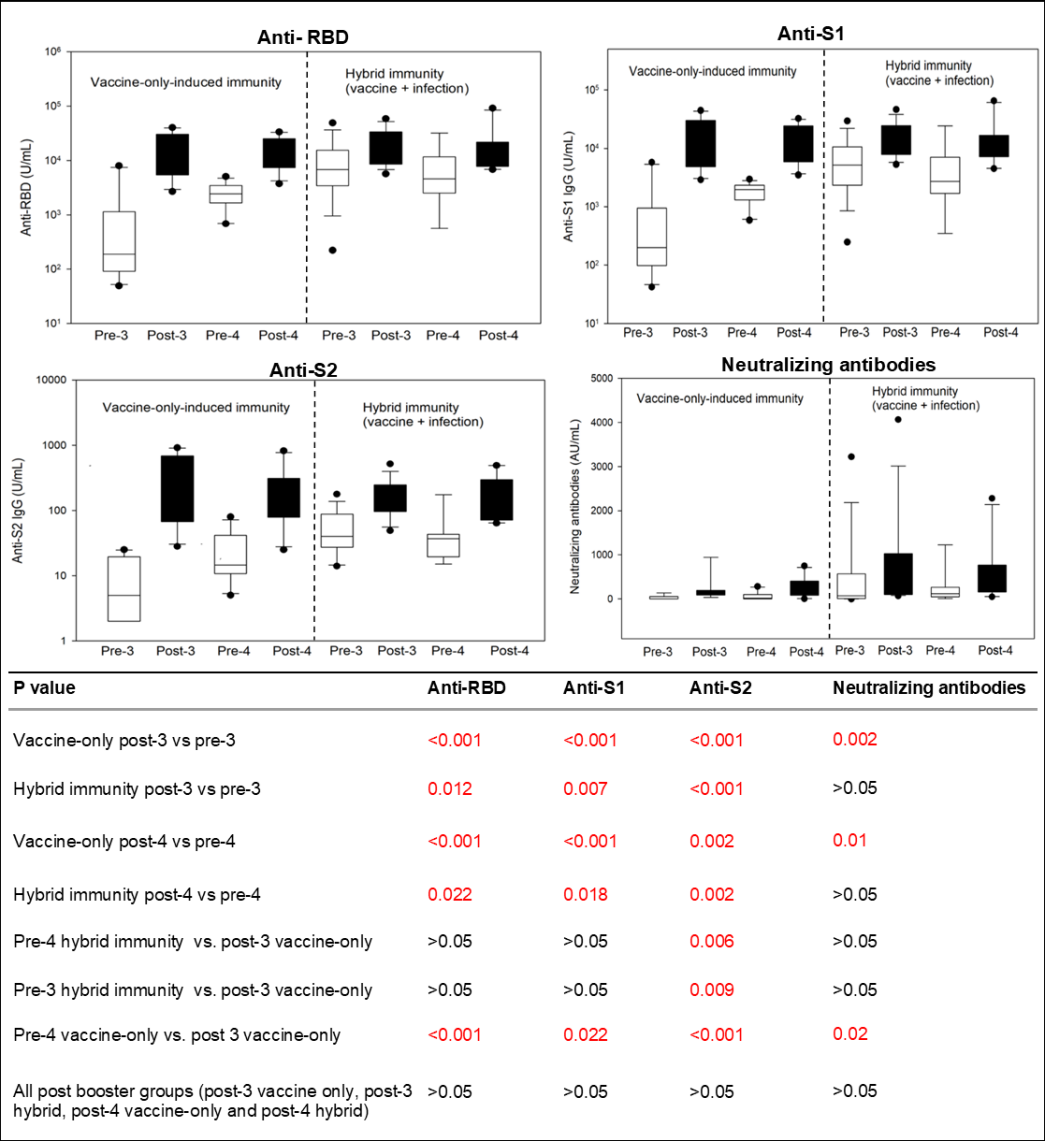
Comparison of SARS-CoV-2 antibodies before and after booster doses of mRNA vaccines. The first booster (third original formula) or second booster (fourth bivalent) dose of mRNA vaccine significantly increases SARS-CoV-2 binding (anti-RBD, anti-S1, and anti-S2) and neutralizing antibodies in individuals with vaccine-only immunity. For individuals with hybrid immunity, their pre-booster (dose 3 or 4) anti-RBD, anti-S1, and neutralizing antibody levels were comparable to vaccine-only individuals’ post-dose-3 antibody levels. After receiving booster doses, there was a further increase in binding antibodies but no significant elevation in neutralizing antibody levels compared to their pre-booster levels. Additionally, there was no significant difference between post-third and post-fourth antibody levels or between hybrid and vaccine-only immunity during 14–28 days after the booster vaccine. Pre-3: prior to the third dose of the original mRNA vaccine. Pre-4: prior to the fourth dose of the bivalent mRNA vaccine. “Hybrid” denotes hybrid immunity due to the combination of vaccination and natural infection. “Vaccine-only” means the participants have been vaccinated and never infected with COVID-19.

Next, we examined the effects of booster vaccines (dose 3 of the original formula and dose 4 of the bivalent vaccine) on the levels of SARS-CoV-2 binding antibodies and neutralizing antibodies (Fig. 2). For participants with vaccine-only immunity, both the post-3 and post-4 SARS-CoV-2 binding antibody levels showed significant increases when compared to their respective pre-booster dose levels. Moreover, the boosters led to significant increases in neutralizing antibody levels for individuals who had only received the vaccine and had not been previously infected. For participants with hybrid immunity, both booster doses (3 or 4) resulted in significantly elevated binding antibody levels. However, neutralizing antibody levels were not significantly changed from their pre-booster levels. Interestingly, despite differences in pre-booster antibody levels between hybrid and vaccine-only immunity or between pre-4 and pre-3, all binding antibodies and neutralizing antibodies reached similar levels after booster administration, regardless of hybrid or vaccine-only immunity or booster doses.

Additionally, for individuals with hybrid immunity, median pre-booster (dose 3 or 4) anti-RBD, anti-S1, and neutralizing antibody levels were comparable to vaccine-only participants’ post-3 antibody levels (Fig. 2). The calculated central 95% intervals of anti-RBD and neutralizing antibodies were 1,921–49,236 U/mL and 10–975 U/mL, respectively, for vaccine-only individuals 14–28 days after receiving their third dose vaccination. Before booster doses, 69% of individuals exceeded the anti-RBD 1,921 U/mL threshold, specifically 20% in the pre-3 vaccine-only, 86% in the pre-3 hybrid immunity, 75% in the pre-4 vaccine-only, and 89% in the pre-4 hybrid immunity group. Similarly, 66% of individuals exceeded the neutralizing antibodies 10 AU/mL threshold, with 20% in the pre-3 vaccine-only, 86% in the pre-3 hybrid immunity, 64% in the pre-4 vaccine-only, and 89% in the pre-4 hybrid immunity group. The agreement between anti-RBD and neutralizing antibodies in assessing whether they exceeded the threshold was 94%.

**Fig 3:**
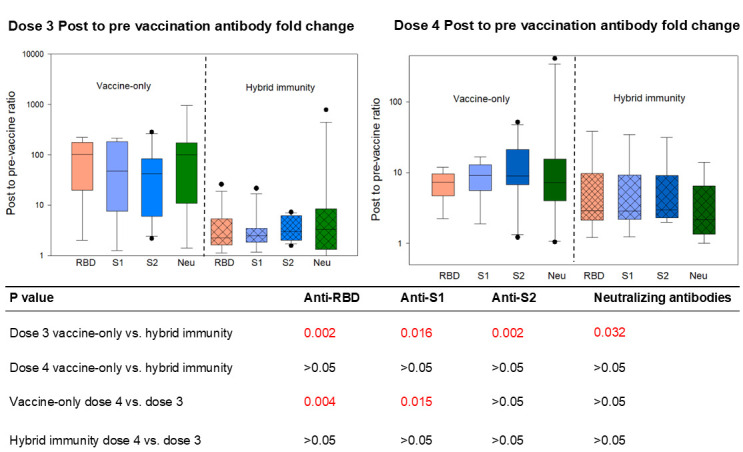
SARS-CoV-2 antibody fold changes after administration of booster doses of mRNA vaccines. Post-booster antibody level fold changes were obtained by calculating the post-3/pre-3 or post-4/pre-4 ratio. The third (first booster) dose vaccine induced a significantly higher fold increase in antibody levels among vaccine-only individuals compared to those with hybrid immunity for both binding and neutralizing antibodies. The fourth (second booster) dose-induced fold change in antibodies was similar between vaccine-only and hybrid immunity. Compared to the fourth bivalent dose, the third original dose induced a higher fold increase in anti-RBD1 and anti-S1 antibodies but not anti-S2 or neutralizing in vaccine-only individuals. The third and fourth dose-induced antibody fold changes were similar for individuals with hybrid immunity. The third dose: i.e., first booster; original mRNA vaccine formula. The fourth dose: i.e., second booster, bivalent mRNA vaccine. Pre-3: prior to the third dose of the original mRNA vaccine. Pre-4: prior to the fourth dose of the bivalent mRNA vaccine. “Hybrid” denotes hybrid immunity due to the combination of vaccination and natural infection. “Vaccine-only” means the participants have been vaccinated and never infected with COVID-19.

We also compared post-booster antibody level fold increase by calculating the post-3/pre-3 or post-4/pre-4 ratio (Fig. 3). The third dose vaccine induced a significantly higher fold increase in antibody levels among vaccine-only participants compared to those with hybrid immunity for both binding and neutralizing antibodies, due to their lower pre-booster levels. However, the fold increase in antibodies induced by the fourth dose was similar between vaccine-only and hybrid immunity participants. Additionally, among participants with vaccine-only immunity, the third original dose induced a higher fold increase in anti-RBD1 and anti-S1 antibodies but not anti-S2 or neutralizing antibodies, compared to the fourth bivalent dose. The third and fourth dose-induced antibody fold changes were similar for participants with hybrid immunity.

**Fig 4:**
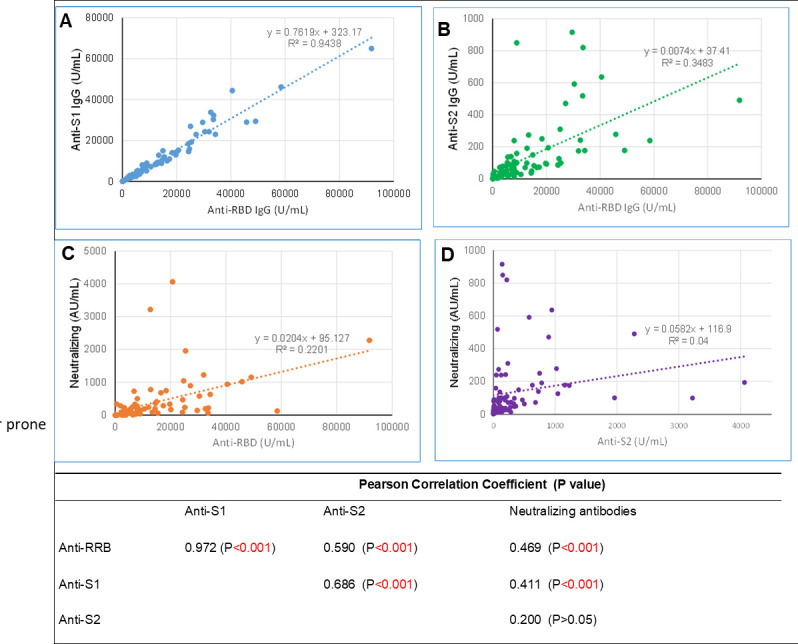
Correlation between binding antibodies against RBD, S1, and S2 and neutralizing antibodies against Omicron BA.2. Anti-RBD and anti-S1 IgG were correlated with neutralizing antibody levels. Anti-RBD exhibited a solid linear regression with anti-S1 IgG but not anti-S2 or neutralizing antibodies. Correlation between anti-S2 and neutralizing antibodies was not observed.

Finally, we assessed the potential correlation between anti-RBD, anti-S1, anti-S2, and neutralizing antibodies and performed regression analyses (Fig. 4). We found that anti-RBD and anti-S1 IgG antibody levels were highly correlated, showing a strong positive linear regression. Additionally, anti-RBD or anti-S1 IgG antibody levels were significantly correlated with neutralizing antibody levels. However, the relationship between these antibodies and neutralizing antibodies did not fit well with either linear or nonlinear regression models. Furthermore, while S2 antibodies showed a correlation with anti-RBD or anti-S1 antibodies, they did not correlate strongly with neutralizing antibodies against BA.2.

## DISCUSSION

In this study, we assessed population immunity levels before and after the first and second booster vaccines, which can provide valuable insights for developing future vaccination strategies. In the United States, healthcare workers were among the early priority groups to complete the primary two doses of the mRNA vaccine around early 2021. In UHHS, many of them then receive their first dose of booster (third dose with the original formula) in late 2021 and a second booster (fourth dose with the bivalent vaccine) in late 2022. Therefore, they are suitable candidates for studying SARS-CoV-2 immunity. Our study showed that 53% of the participants had hybrid immunity based on either positive anti-nucleocapsid results or self-reported PCR results by December 2022. If based on anti-nucleocapsid alone, 40% had hybrid immunity, and 13% would be miscategorized. The hybrid immunity rate in the current study is similar to the reported 47.7% hybrid immunity ascertained by the presence of anti-nucleocapsid antibodies and anti-spike antibodies in 142,758 blood donors between April 2021 and September 2022 (6).

Many studies have investigated the longevity of SARS-CoV-2 antibodies following primary vaccination or infection and have developed estimation models (7–9). However, few studies have evaluated the duration of antibodies following booster vaccines. Additionally, most existing studies have focused on antibodies against the index (also known as the original) strain or Omicron BA.1. As real-world scenarios continue to diversify, encompassing factors such as times of repeated infections, doses of booster vaccines, viral variants, and vaccine types, predicting antibody longevity without measurement is extremely difficult if not impossible. We quantitated anti-RBD, S1, S2, and neutralizing antibodies against Omicron BA.2, which the recent prevalence strains (JN.1, HV.1, XBB.1, BA.4, and BA.5, https://covid.cdc.gov/covid-data-tracker/#variant-proportions) derived from. Our study demonstrated that anti-RBD/S1 binding antibodies persisted in all participants regardless of whether they had hybrid or vaccine-only immunity but varied greatly in concentration 1 year after completing the primary mRNA vaccine series. The antibody levels were notably lower in the vaccine-only individuals than those with hybrid immunity but higher than our previously reported antibody levels in unvaccinated healthcare workers who recovered from infection with the index strain (10). Moreover, more than 90% of participants with hybrid immunity also retained neutralizing antibodies at variable levels, whereas most vaccine-only participants did not have detectable neutralizing antibodies. Around 1 year after the first dose of the booster, all participants still exhibited detectable levels of neutralizing antibodies, indicating a lower waning rate after the booster vaccine. This finding aligns with Jacobsen et al.’s (7) findings that post-booster neutralizing antibody reduction from months 1 to 6 against the index strain was slower than the post-primary series reduction.

The significance of residual antibody levels, i.e., whether or not protective from severe disease or death, remains to be determined. A systematic review (3) showed that previous infection can protect against hospital admission or severe disease with 74.6% effectiveness at 12 months but only 24.7% against reinfection. Similarly, hybrid immunity at 12 months can offer 97.4% effectiveness against hospital admission or severe disease with primary series vaccination and 41.8% against reinfection. The persistence of neutralizing antibodies in combination with anti-RBD antibodies greater than 1,000 U/mL in over 90% of individuals with hybrid immunity in our study is consistent with these findings. Individuals with hybrid immunity, possessing high levels of neutralizing and anti-RBD antibodies, may benefit from significant protection against new variants originating from Omicron BA.2, due to cross-protection. For instance, a study conducted by Stamatatos et al. (11) discovered that individuals with hybrid immunity exhibited higher neutralizing antibody titers against multiple variants compared to those with immunity from vaccination or natural infection alone. Feng et al. (12) estimated a vaccine effectiveness of 80% against symptomatic infection with the majority Alpha (B.1.1.7) variant of SARS-CoV-2 was achieved with 506 BAU/mL for anti-spike and anti-RBD antibodies. Based on this threshold, 93% of hybrid immunity and 40% of vaccine-only individuals retained such protective levels approximately 1 year after their initial two-dose vaccination. Furthermore, 100% of individuals maintained a protective level of anti-RBD antibodies 1 year after receiving the first booster dose of mRNA vaccines. The Omicron variant of SARS-CoV-2 presents challenges to vaccine efficacy due to its numerous mutations. As a result, higher levels of anti-spike (anti-S) and anti-receptor binding domain (anti-RBD) antibodies may be required to ensure sufficient protection against infection. Studies have indicated that increased levels of these antibodies are linked to greater protection against symptomatic COVID-19, particularly with variants such as Omicron (https://www.medrxiv.org/content/10.1101/2022.11.29.22282916v1). It should be noted that different assays used for measuring binding and neutralizing antibodies also contribute to the larger variation in the estimated protection antibody levels. Even after standardizing measurements using the WHO standards to the international unit, neutralization titers across the assays can still be different up to 50-fold (8). Taken together, a well-accepted threshold for protection had not been established for either binding or neutralizing antibodies. The noticeable concentration of residual anti-RBD and anti-S1 antibodies in individuals with hybrid immunity and those who received booster vaccines in our study suggests potential protection. However, definitive determination is hindered by the absence of a universally accepted threshold.

Furthermore, we interpret whether residual antibody levels are adequate or necessitate a booster by comparing them to post-booster antibody levels. The median neutralizing antibodies, anti-RBD, and anti-S1 binding antibodies in subjects with hybrid immunity before booster doses were not statistically different from the vaccine-only subjects’ post-booster levels. Additionally, over 80% of individuals with hybrid immunity demonstrated pre-booster residual anti-RBD and neutralizing antibodies above the vaccine-only post-first booster central 95% threshold. These findings suggest that for most immunocompetent individuals with hybrid immunity, anti-RBD and neutralizing antibodies may persist over 1 year at levels similar to those seen 14–28 days after the first booster in vaccine-only individuals. Further research is needed to determine the optimal timing for additional booster doses for individuals with hybrid immunity. Vaccine-only individuals also retained significant amounts of binding antibodies against RBB and S1, but only 30% had detectable neutralizing antibodies, and only 20% exceeded the vaccine-only post-first booster central 95% threshold 1 year after the primary vaccine series, suggesting a booster will benefit most individuals who completed the primary vaccine series but were never infected. One year after the first booster, vaccine-only individuals retained about 10 times higher levels of binding antibodies than after the primary vaccine series, all retained detectable neutralizing antibodies, and 64% exceeded the vaccine-only post-first booster central 95% threshold. These findings demonstrated a booster dose is beneficial. Importantly, booster vaccines bridged the gap between vaccine-only and hybrid immunity, bringing up antibodies of vaccine-only individuals to the same post-vaccination level as those with hybrid immunity. Both binding and neutralizing antibodies after administering the first or second booster were equivalent for all participants despite the large pre-booster difference between vaccine-only and hybrid immunity. Booster vaccines are important for maintaining immunity against emerging variants such as Omicron BA.2. They improve the immune response by targeting variant-specific epitopes, helping to sustain population immunity and adapt to the changing threat landscape. These data suggest that booster vaccines can offer the same level of protection as hybrid immunity, further emphasizing the need for at least one booster for those with vaccine-only immunity.

Our study also demonstrated that booster doses significantly increased both binding and neutralizing antibody levels in all subjects, but to a much lesser degree for individuals with high pre-booster antibody concentrations, as evidenced by the significantly smaller fold change compared to those with low pre-booster levels. The bivalent vaccine boosted the anti-SARS-CoV-2 antibodies similarly to the original vaccine. The smaller increase in anti-RBD and anti-S1 among vaccine-only individuals after the second booster compared to the first booster dose is likely due to higher pre-booster concentrations rather than differences in the booster vaccine formula. These findings question the necessity of annual boosters for those with high antibody levels, especially with hybrid immunity, and emphasize the importance of considering an individual’s vaccination and infection history when planning vaccination strategies.

Neutralizing antibody titer is considered the gold standard for correlating immunity to protection. Despite the development of various surrogate assays to replace the conventional virus neutralization test for detecting anti-SARS-CoV-2 neutralizing antibodies, these surrogate assays still require serial dilutions to measure titers semi-quantitatively. This process is typically manual, labor-intensive, and error-prone. Anti-RBD or anti-S1 binding antibody assays are widely available on various automated immunoassay platforms, many of which have been approved by the FDA for *in vitro* diagnosis. Numerous studies have shown that anti-RBD and anti-S1 binding antibody concentrations correlate with neutralizing antibody levels against the index strain and have explored different regression models (13, 14). Given the evolving nature of the virus but the unchanged antigen from the initial index strain in most commercially available binding assays, the question arises: does the correlation remain? Our study demonstrated binding antibody assays against index strain RBD or S1 remain strongly correlated with neutralizing antibodies against the BA.2 strain from which the recent popular stains are derived. However, accurate estimation of neutralizing antibodies using regression models is difficult to achieve. Instead, establishing a binding antibody cutoff for detectable neutralizing antibodies may be possible. Our data showed that neutralizing antibodies were undetectable when anti-RBD antibodies were <250 U/mL. Larger-scale studies are needed to refine this cutoff level further.

### Limitation

It should be noted that the study included mainly adults under the age of 65 who did not have any immunocompromising conditions. Hence, the findings and interpretation cannot be generalized to the elderly, children, or immunocompromised individuals. The relatively small sample size may not be sufficiently powered to detect a difference between the groups and, thus, may be prone to a type II error. Furthermore, there were considerable variations in neutralizing and binding antibody levels between participants with hybrid immunity, indicating that the overall conclusion may not be applicable to every individual. Additionally, the detection of neutralizing antibodies was limited to the BA.2 variant using the GenScript assay, and the findings may not apply to variants distant from BA.2. Future longitudinal studies are needed to evaluate the long-term durability of humoral immunity following booster vaccinations against new dominant variants.

### Conclusion

Four years into the COVID-19 pandemic, the population has gained significant but varying degrees of immunity, evidenced by variable levels of neutralizing antibodies against Omicron BA.2 and binding antibodies against RBD, S1, and S2. Individuals with hybrid immunity retain a notable amount of antibodies beyond 1 year after vaccination. Therefore, vaccination planning needs to consider both vaccination and infection history. Further investigation is warranted to determine the optimal timing for subsequent booster doses. On the other hand, booster vaccines have helped bridge the gap between vaccine-only and hybrid immunity and are beneficial for increasing antibody levels to ensure sufficient protection against infection.
